# Supplementary material for: The impact of data resolution on dynamic causal inference in multiscale ecological networks
Source: Commun Biol. 2024 Nov 5;7:1442. doi: 10.1038/s42003-024-07054-z (PMC11538442; doi:10.1038/s42003-024-07054-z)
Supplement: Supplementary file 2 — Supplementary Information [file 42003_2024_7054_MOESM2_ESM.pdf]

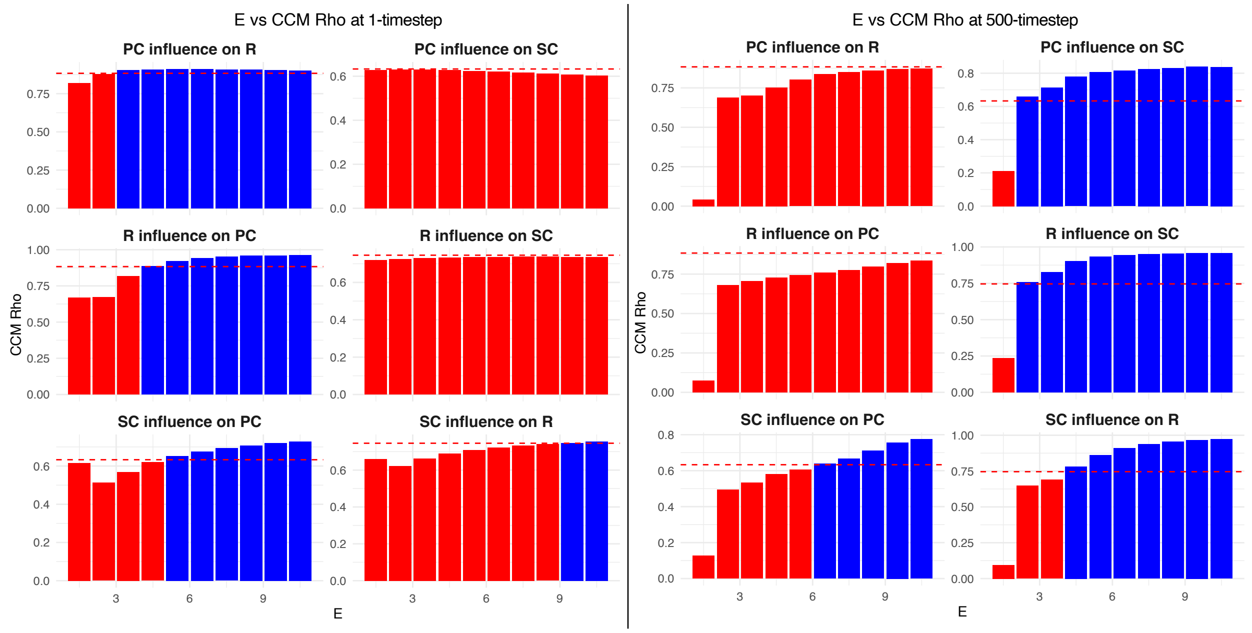

Figure S1. Performing CCM in the IBM model with varying embedding dimensions. Red bars signify a non-significant causal interaction (CCM rho < absolute cross correlation). Blue bars signify that a significant relationship was identified. Left charts show CCM computed with tau = 1 and right plots show CCM with tau = 500.

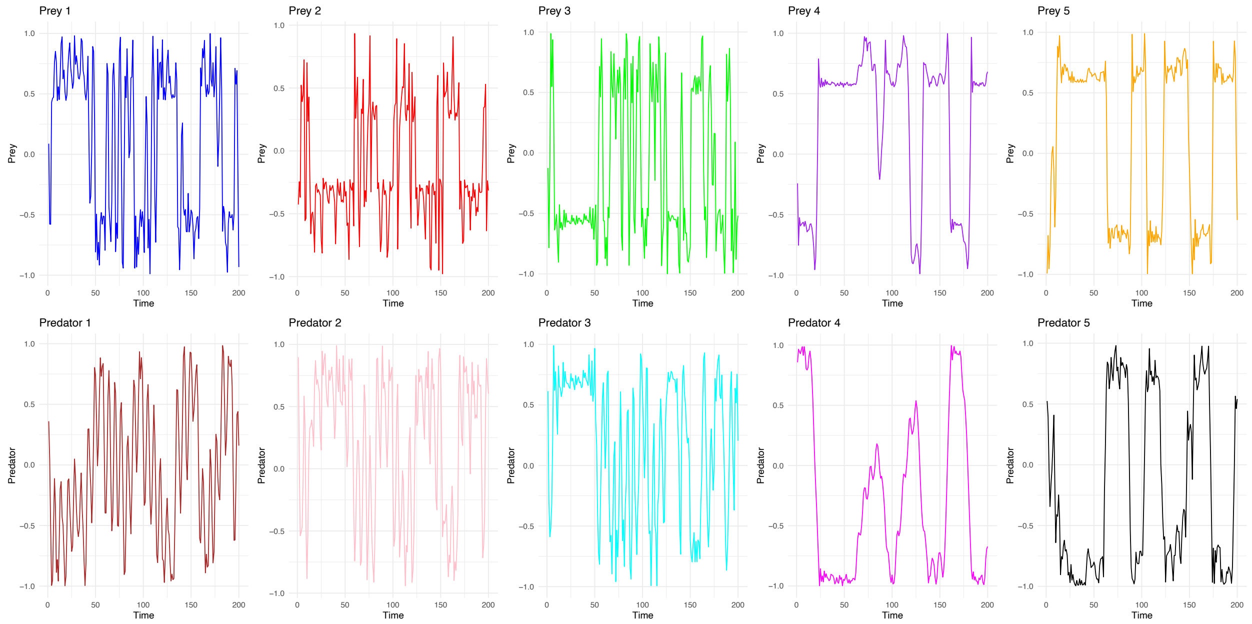

Figure S2. Examples of timeseries generated from the coupled logistic models.

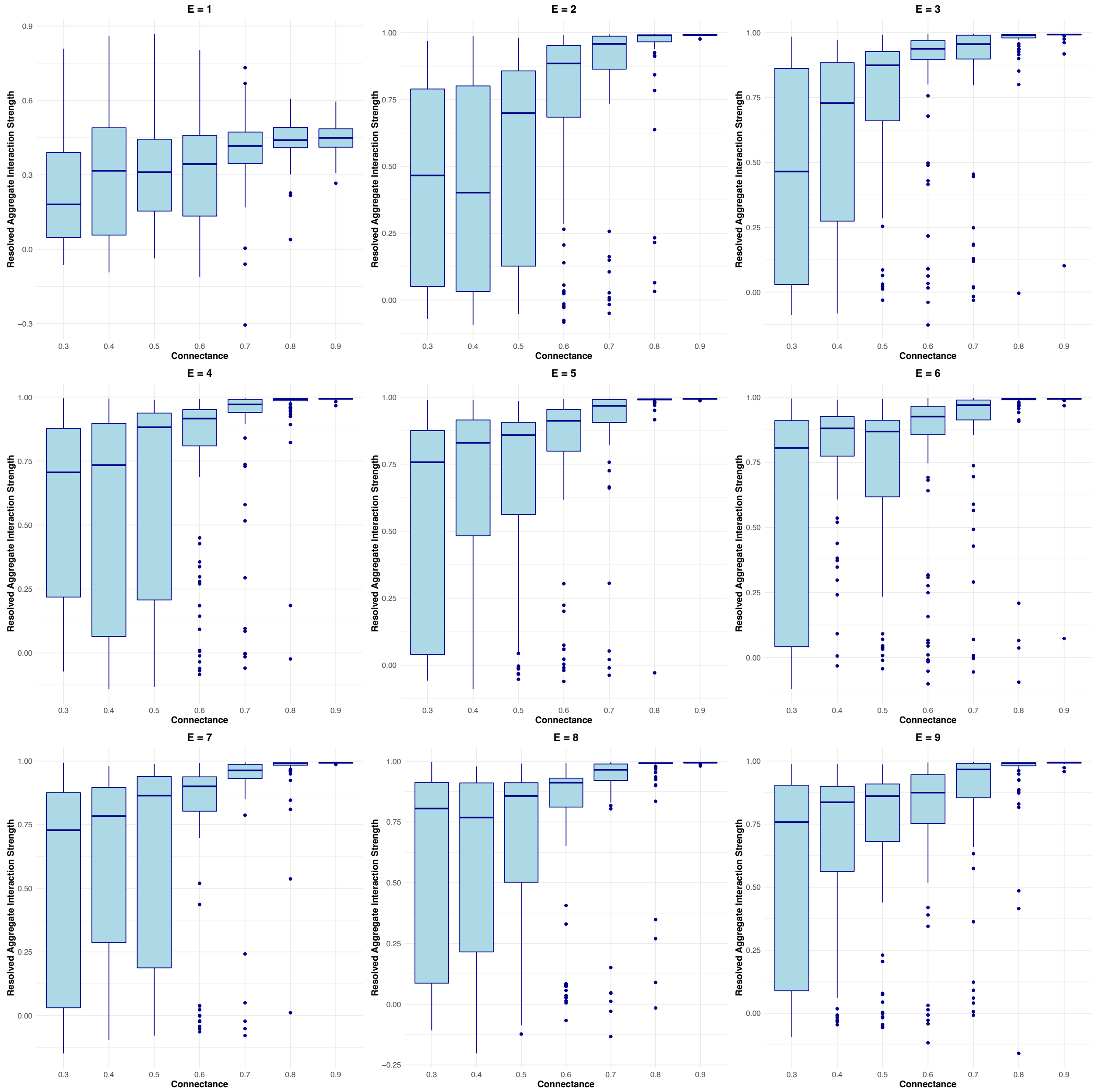

Figure S3. Analogous to Fig. 3, but computed at varying embedding dimensions. Values represent boxplot medians for that embedding dimension.

Table S1. The list of species and functional groups with their corresponding number of monthly resolved interactions and annual resolved interactions for the North Sea, Lake Zurich, and Port Erin Bay systems. Highlights show species mentioned in the main text.

System	Functional Group	Species	Monthly Interactions	Annual Interactions
Lake Zurich	Diatoms	Thalassiosirales Stephanodiscus sp	29	9
Lake Zurich	Diatoms	Centrales	36	8
Lake Zurich	Diatoms	Tabellariales Asterionella formosa	16	6
Lake Zurich	Diatoms	Cymbellales Cymbella sp	35	9
Lake Zurich	Diatoms	Fragilariales Fragilaria crotonensis	18	5
Lake Zurich	Diatoms	Bacillariales Nitzschia sp	32	9
Lake Zurich	Diatoms	Licmophorales Ulnaria delicatissima var angustissima	14	4
Lake Zurich	Diatoms	Tabellariales Tabellaria fenestrata	19	5
Lake Zurich	Dinoflagellates	Gymnodiniales Gymnodinium sp	26	7
Lake Zurich	Dinoflagellates	Gymnodiniales Gymnodinium helveticum	27	8
Lake Zurich	Dinoflagellates	Gonyaulacales Ceratium hirundinella	37	4
Lake Zurich	Cryptophytes	Pyrenomonadales Rhodomonas sp	37	9

System	Functional Group	Species	Monthly Interactions	Annual Interactions
Lake Zurich	Green Algae	Volvocales Phacotus lenticularis	36	5
Lake Zurich	Green Algae	Volvocales Pandorina morum	26	7
Lake Zurich	Green Algae	Klebsormidiales Elakatothrix gelatinosa	15	10
Lake Zurich	Cyanobacteria	Nostocales Aphanizomenon flos-aquae	19	3
Lake Zurich	Cyanobacteria	Oscillatoriales Planktothrix rubescens	28	18
Lake Zurich	Green Algae	Desmidiales Closterium aciculare	19	12
Lake Zurich	Green Algae	Desmidiales Cosmarium sp	21	4
Lake Zurich	Green Algae	Desmidiales Staurastrum sp	32	24
Lake Zurich	Gold Algae	Chromulinales Dinobryon sp	23	4
Lake Zurich	Herbivore Cladocerans	Diplostraca Bosmina longirostris adult	32	21
Lake Zurich	Herbivore Cladocerans	Diplostraca Bosmina longirostris juvenile	31	17
Lake Zurich	Herbivore Cladocerans	Diplostraca Bosmina longirostris juvenile	28	19

System	Functional Group	Species	Monthly Interactions	Annual Interactions
Lake Zurich	Calanoid Copepods	Calanoida Diaptomus sp eggs	27	13
Lake Zurich	Calanoid Copepods	Calanoida Diaptomus sp C1-C3	26	14
Lake Zurich	Calanoid Copepods	Calanoida Diaptomus sp C4-C5	21	13
Lake Zurich	Calanoid Copepods	Calanoida Eudiaptomus gracilis male	13	16
Lake Zurich	Calanoid Copepods	Calanoida Eudiaptomus gracilis ovaria	10	8
Lake Zurich	Calanoid Copepods	Calanoida Eudiaptomus gracilis female	15	14
Lake Zurich	Calanoid Copepods	Calanoida Eudiaptomus gracilis male	12	14
Lake Zurich	Cyclopoid Copepods	Cyclopoida Cyclops abyssorum male	19	15
Lake Zurich	Cyclopoid Copepods	Cyclopoida Cyclops sp SU C1-C3	34	13
Lake Zurich	Cyclopoid Copepods	Cyclopoida Cyclops sp SU C4-C5	26	16
Lake Zurich	Cyclopoid Copepods	Cyclopoida nauplia	32	13
Lake Zurich	Cyclopoid Copepods	Cyclopoida Cyclops abyssorum female	9	14

System	Functional Group	Species	Monthly Interactions	Annual Interactions
Lake Zurich	Cyclopoid Copepods	Cyclopoida Cyclops abyssorum male	8	9
Lake Zurich	Cyclopoid Copepods	Cyclopoida Cyclops sp eggs	24	10
Lake Zurich	Herbivore Cladocerans	Diplostraca eggs	35	8
Lake Zurich	Gold Algae	Ochromonadales Erkenia subaequiciliata	27	3
North Sea	Larvae	Appendicularia	13	3
North Sea	Diatoms	Asterionellopsis glacialis	8	8
North Sea	Diatoms	Bacillaria paxillifera	4	7
North Sea	Diatoms	Bacteriastrum spp.	14	6
North Sea	Larvae	Bivalvia larvae	11	3
North Sea	Omnivorous Copepod	Calanus finmarchicus	17	7
North Sea	Omnivorous Copepod	Calanus helgolandicus	15	7
North Sea	Omnivorous Copepod	Calanus I-IV	12	2
North Sea	Omnivorous Copepod	Candacia armata	19	8
North Sea	Omnivorous Copepod	Centropages hamatus	8	1
North Sea	Omnivorous Copepod	Centropages typicus	16	1
North Sea	Dinoflagellate	Ceratium furca	20	4
North Sea	Dinoflagellate	Ceratium fusus	18	2
North Sea	Dinoflagellate	Ceratium horridum	12	5

System	Functional Group	Species	Monthly Interactions	Annual Interactions
North Sea	Dinoflagellate	Ceratium lineatum	19	4
North Sea	Dinoflagellate	Ceratium longipes	15	4
North Sea	Dinoflagellate	Ceratium macroceros	20	7
North Sea	Dinoflagellate	Ceratium tripos	21	4
North Sea	Diatoms	Chaetoceros(Hyalochaete) spp.	11	4
North Sea	Diatoms	Chaetoceros(Phaeoceros) spp.	17	4
North Sea	Larvae	Cirripede larvae (Total)	17	6
North Sea	Larvae	Clione limacina	16	5
North Sea	Omnivorous Copepod	Copepod eggs	28	3
North Sea	Omnivorous Copepod	Copepod nauplii	11	3
North Sea	Diatoms	Corethron hystrix	12	2
North Sea	Omnivorous Copepod	Corycaeus spp.	11	12
North Sea	Larvae	Cumacea	7	5
North Sea	Larvae	Cyphonautes	6	2
North Sea	Larvae	Decapoda larvae (Total)	19	1
North Sea	Dinoflagellate	Dinophysis spp. Total	13	2
North Sea	Diatoms	Ditylum brightwellii	8	4
North Sea	Larvae	Echinoderm larvae	18	2
North Sea	Larvae	Euphausiacea calyptopis	10	2
North Sea	Larvae	Evadne spp.	20	2

System	Functional Group	Species	Monthly Interactions	Annual Interactions
North Sea	Fish	Fish larvae	17	2
North Sea	Diatoms	Fragilaria spp.	8	7
North Sea	Larvae	Gammaridea	11	3
North Sea	Dinoflagellate	Gonyaulax spp.	13	4
North Sea	Omnivorous Copepod	Harpacticoida Total Traverse	11	2
North Sea	Larvae	Hyperiidea (Total)	13	5
North Sea	Omnivorous Copepod	Labidocera wollastoni	10	4
North Sea	Diatoms	Leptocylindrus danicus	4	5
North Sea	Omnivorous Copepod	Metridia lucens	11	4
North Sea	Larvae	Mysidacea	6	6
North Sea	Diatoms	Odontella aurita	9	8
North Sea	Diatoms	Odontella sinensis	9	6
North Sea	Herbivorous Copepod	Oithona spp.	19	2
North Sea	Omnivorous Copepod	Para-Pseudocalanus spp.	21	2
North Sea	Diatoms	Paralia sulcata	16	11
North Sea	Larvae	Podon spp.	7	4
North Sea	Diatoms	Proboscia alata	14	5
North Sea	Diatoms	Proboscia inermis	5	4
North Sea	Dinoflagellate	Protoperidinium spp.	18	2
North Sea	Diatoms	Pseudo-nitzschia delicatissima complex	9	4

System	Functional Group	Species	Monthly Interactions	Annual Interactions
North Sea	Diatoms	Pseudo-nitzschia seriata complex	12	3
North Sea	Herbivorous Copepod	Pseudocalanus spp. Adult Atlantic	12	4
North Sea	Diatoms	Rhizosolenia hebetata semispina	14	4
North Sea	Diatoms	Rhizosolenia imbricata	13	5
North Sea	Diatoms	Rhizosolenia setigera	10	6
North Sea	Diatoms	Rhizosolenia styliformis	12	6
North Sea	Diatoms	Skeletonema costatum	12	4
North Sea	Omnivorous Copepod	Temora longicornis	9	1
North Sea	Diatoms	Thalassionema nitzschioides	18	5
North Sea	Diatoms	Thalassiosira spp.	16	6
North Sea	Diatoms	Thalassiothrix longissima	8	6
North Sea	Larvae	Thecosomata (North Atlantic)	12	5
North Sea	Larvae	Tomopteris spp.	8	6
Port Erin Bay	Herbivorous Copepod	Acartia.clausi	16	2
Port Erin Bay	Diatoms	Biddulphia.mobiliensis	9	2
Port Erin Bay	Omnivorous Copepod	Calanus.finmarchicus	10	2
Port Erin Bay	Omnivorous Copepod	Centropages.hamatus	12	3

System	Functional Group	Species	Monthly Interactions	Annual Interactions
Port Erin Bay	Dinoflagellate	Ceratium.tripos	14	3
Port Erin Bay	Diatoms	Chaetoceras.debile	7	1
Port Erin Bay	Diatoms	Chaetoceras.decipiens	11	5
Port Erin Bay	Diatoms	Chaetoceras.sociale	5	3
Port Erin Bay	Diatoms	Chaetoceras.teres	6	4
Port Erin Bay	Diatoms	Coscinodiscus.concinnus	13	1
Port Erin Bay	Diatoms	Coscinodiscus.grani	11	1
Port Erin Bay	Diatoms	Coscinodiscus.radiatus	10	2
Port Erin Bay	Larvae	Echinoderm.plutei	13	2
Port Erin Bay	Larvae	Gastropod.larvae	12	3
Port Erin Bay	Diatoms	Guinardia.flaccida	8	2
Port Erin Bay	Larvae	Lamellibranch.larvae	13	2
Port Erin Bay	Diatoms	Lauderia.borealis	5	1
Port Erin Bay	Herbivorous Copepod	Oithona.similis	15	2
Port Erin Bay	Herbivorous Copepod	Paracalanus.parvus	9	3
Port Erin Bay	Larvae	Polychaete.larvae	15	1
Port Erin Bay	Herbivorous Copepod	Pseudocalanus.elongatus	9	2
Port Erin Bay	Diatoms	Rhizosolenia.semispina	4	1
Port Erin Bay	Diatoms	Rhizosolenia.setigera	4	3

System	Functional Group	Species	Monthly Interactions	Annual Interactions
Port Erin Bay	Diatoms	Rhizosolenia.shrubsolei	8	4
Port Erin Bay	Diatoms	Rhizosolenia.stolterfothii	5	1
Port Erin Bay	Chaetagnath	Sagitta.bipunctata	6	2
Port Erin Bay	Omnivorous Copepod	Temora.longicornis	12	2
Port Erin Bay	Diatoms	Thalassiosira.nordenskioldi	5	1
