# Supplementary material for: The impact of data resolution on dynamic causal inference in multiscale ecological networks
Source: Commun Biol. 2024 Nov 5;7:1442. doi: 10.1038/s42003-024-07054-z (PMC11538442; doi:10.1038/s42003-024-07054-z)
Supplement: Supplementary file 3 — Reporting Summary [file 42003_2024_7054_MOESM3_ESM.pdf]

nature portfolio

Corresponding author(s): Erik Saberski

Last updated by author(s): 9/23/2024

Reporting Summary

Nature Portfolio wishes to improve the reproducibility of the work that we publish. This form provides structure for consistency and transparency in reporting. For further information on Nature Portfolio policies, see our Editorial Policies and the Editorial Policy Checklist.

Statistics

For all statistical analyses, confirm that the following items are present in the figure legend, table legend, main text, or Methods section.

n/a	Confirmed	
☐	☒	The exact sample size (*n*) for each experimental group/condition, given as a discrete number and unit of measurement
☐	☒	A statement on whether measurements were taken from distinct samples or whether the same sample was measured repeatedly
☐	☒	The statistical test(s) used AND whether they are one- or two-sided<br>*Only common tests should be described solely by name; describe more complex techniques in the Methods section.*
☐	☒	A description of all covariates tested
☐	☒	A description of any assumptions or corrections, such as tests of normality and adjustment for multiple comparisons
☐	☒	A full description of the statistical parameters including central tendency (e.g. means) or other basic estimates (e.g. regression coefficient) AND variation (e.g. standard deviation) or associated estimates of uncertainty (e.g. confidence intervals)
☐	☒	For null hypothesis testing, the test statistic (e.g. *F*, *t*, *r*) with confidence intervals, effect sizes, degrees of freedom and *P* value noted<br>*Give P values as exact values whenever suitable.*
☐	☒	For Bayesian analysis, information on the choice of priors and Markov chain Monte Carlo settings
☐	☒	For hierarchical and complex designs, identification of the appropriate level for tests and full reporting of outcomes
☐	☒	Estimates of effect sizes (e.g. Cohen's *d*, Pearson's *r*), indicating how they were calculated

Our web collection on statistics for biologists contains articles on many of the points above.

Software and code

Policy information about availability of computer code

Data collection	Data from four publically available monitoring studies were used in this analysis:<br>1) The North Sea from the Survey of the Marine Biological Association, formerly the Sir Alister Hardy Foundation for Ocean Science (SAHFOS),<br>2) Port Erin Bay from MetaBase,<br>3) Lake Zurich from Pomati et al. 2020,<br>4) A kelp forest system of San Nicholas Island from Kenner et al. 2013.
Data analysis	The EDM analyses were performed using the R package rEDM version 0.7.5.

For manuscripts utilizing custom algorithms or software that are central to the research but not yet described in published literature, software must be made available to editors and reviewers. We strongly encourage code deposition in a community repository (e.g. GitHub). See the Nature Portfolio guidelines for submitting code & software for further information.

Data

Policy information about availability of data

All manuscripts must include a data availability statement. This statement should provide the following information, where applicable:

- Accession codes, unique identifiers, or web links for publicly available datasets
- A description of any restrictions on data availability
- For clinical datasets or third party data, please ensure that the statement adheres to our policy

All data and scripts used to perform analyses are made publicly available at https://github.com/SugiharaLab/Data-Resolution-Causal-Inference

Research involving human participants, their data, or biological material

Policy information about studies with human participants or human data. See also policy information about sex, gender (identity/presentation), and sexual orientation and race, ethnicity and racism.

Reporting on sex and gender	N/A
Reporting on race, ethnicity, or other socially relevant groupings	N/A
Population characteristics	N/A
Recruitment	N/A
Ethics oversight	N/A

Note that full information on the approval of the study protocol must also be provided in the manuscript.

Field-specific reporting

Please select the one below that is the best fit for your research. If you are not sure, read the appropriate sections before making your selection.

☐ Life sciences ☐ Behavioural & social sciences ☒ Ecological, evolutionary & environmental sciences

For a reference copy of the document with all sections, see nature.com/documents/nr-reporting-summary-flat.pdf

Ecological, evolutionary & environmental sciences study design

All studies must disclose on these points even when the disclosure is negative.

Study description	This study focuses on a combination of simulated model data and real-world species abundance times-eries data spanning four aquatic ecosystems and many taxa. Each of the four natural systems studied here were collected by separate groups and the data is publicly available. We performed Convergenct Cross-mapping (CCM) on these time-series to identify causal relationships between species.
Research sample	The natural systems studied here are from the following sources:<br>1) The North Sea from the Survey of the Marine Biological Association, formerly the Sir Alister Hardy Foundation for Ocean Science (SAHFOS),<br>2) Port Erin Bay from MetaBase,<br>3) Lake Zurich from Pomati et al. 2020,<br>4) A kelp forest system of San Nicholas Island from Kenner et al. 2013.<br><br>These datasets were chosen due to their extensive length of monitoring and coverage of many species.
Sampling strategy	Each system had a different sampling strategy (see Data Collection). Although the sampling strategy varied across systems, the general goal of identifying causal relationships using CCM if robust to sampling strategy used - as long as the sampling strategy was consistent within each system.
Data collection	Each system had a different sampling strategy:<br>1) The North Sea dataset is collected from Continuous Plankton Recorders towed by ships.<br>2) Port Erin sampling is described here: https://www.st.nmfs.noaa.gov/copepod/time-series/uk-30701/<br>3) Lake Zurich data was collected consistent sampling and counting by experts (see Pomati, Francesco, et al. "Interacting temperature, nutrients and zooplankton grazing control phytoplankton size-abundance relationships in eight Swiss lakes." Frontiers in microbiology 10 (2020): 510720.)<br>4) The kelp forest system was monitored using permanent transects (see Kenner, Michael C., et al. "A multi-decade time series of kelp forest community structure at San Nicolas Island, California." Ecology 94.11 (2013): 26-54.)

Timing and spatial scale	1) North Sea:<br>Start: 1958-01-01<br>End: 2013-12-01<br>Sampled monthly across the entire North Sea<br><br>2) Port Erin Bay<br>Start: 2007-01-01<br>End: 2020-12-01<br>Sampled Monthly from a single station<br><br>3) Lake Zurich<br>Start: 1977-01-01<br>End: 2008-12-01<br>Sampled monthly from a consistent sampling location and depth.<br><br>4) Kelp Forest:<br>Start: 1982-06-01<br>End: 2010-06-01<br>Sampled bianually from permanent transects but computed as annual averages.
Data exclusions	To ensure quality and uniformity in the analyses for each dataset, taxa whose time series contained less than 35 non-zero data points or were known to be inconsistently monitored were removed from the analysis.
Reproducibility	N/A
Randomization	Species were organized into their approriate taxanomic aggregates (e.g, "diatoms" and "omnivorous copepods").
Blinding	Blinding was not necessary for the sampling used in this study.

Did the study involve field work? ☐ Yes ☒ No

Reporting for specific materials, systems and methods

We require information from authors about some types of materials, experimental systems and methods used in many studies. Here, indicate whether each material, system or method listed is relevant to your study. If you are not sure if a list item applies to your research, read the appropriate section before selecting a response.

Materials & experimental systems

n/a	Involved in the study
☒	☐ Antibodies
☒	☐ Eukaryotic cell lines
☒	☐ Palaeontology and archaeology
☒	☐ Animals and other organisms
☒	☐ Clinical data
☒	☐ Dual use research of concern
☒	☐ Plants

Methods

n/a	Involved in the study
☒	☐ ChIP-seq
☒	☐ Flow cytometry
☒	☐ MRI-based neuroimaging

Plants

Seed stocks	N/A
Novel plant genotypes	N/A
Authentication	N/A
